# Epidemiological Features of Infantile Hypertrophic Pyloric Stenosis in Taiwanese Children: A Nation-Wide Analysis of Cases during 1997–2007

**DOI:** 10.1371/journal.pone.0019404

**Published:** 2011-05-03

**Authors:** Mee-Mee Leong, Solomon Chih-Cheng Chen, Chih-Sung Hsieh, Yow-Yue Chin, Teck-Siang Tok, Shu-Fen Wu, Ching-Tien Peng, An-Chyi Chen

**Affiliations:** 1 Department of Pediatrics, Pingtung Christian Hospital, Pingtung, Taiwan; 2 Children Medical Center, China Medical University Hospital, Taichung, Taiwan; 3 College of Medicine, Taipei Medical University, Taipei, Taiwan; University of Florida, United States of America

## Abstract

**Objective:**

To describe the epidemiological characteristics of infantile hypertrophic pyloric stenosis (IHPS) in ethnic Chinese children.

**Materials and Methods:**

We reviewed the National Health Insurance claims database and analyzed data from children less than one year of age who had been diagnosed with IHPS (ICD-9-CM 750.5) and had undergone pyloromyotomy (ICD-9-CM 43.3). We analyzed the incidence, gender, age at diagnosis, length of hospital stay, seasonal variation and cost of IHPS from data collected between January 1997 and December 2007.

**Results:**

A total of 1,077 infants met inclusion criteria, including 889 boys and 188 girls. The annual incidence of IHPS ranged from 0.30 to 0.47 per 1,000 live births with a mean incidence of 0.39 per 1,000 live births. Between 2002 and 2007, the incidence showed a declining trend (*P* = 0.025) with coincidentally increasing trends for both exclusive breastfeeding (*P* = 0.014) and breastfeeding plus bottle feeding (*P* = 0.004). The male-to-female rate ratio was dynamic and increased from 3.03 during the first two weeks of life to 8.94 during the 8^th^ through 10^th^weeks of life. The overall male-to-female rate ratio was 4.30. The mean age at diagnosis was 43.1±2.4 days. After analyzing the months of birth and hospital admission, no seasonal variation associated with IHPS was detected. The mean length of hospital stay was 8.28±7.10 days.

**Conclusions:**

The incidence of IHPS in Taiwan, a country with a majority ethnic Chinese population, was lower than observed incidences in Caucasian populations living in Western countries. Breastfeeding campaigns and low maternal smoking rates may contribute to the lower incidence of IHPS in Taiwan. However, additional studies with longer follow-up periods are needed.

## Introduction

Infantile hypertrophic pyloric stenosis (IHPS) is an abnormal hypertrophy of the muscle at the pylorus that results in gastric outlet obstruction. However, its etiology is still unknown. It commonly affects infants younger than one year of age. The classic presentations of IHPS are projectile vomiting immediately following feeding and a palpable abdominal mass in the upper abdomen [1]. Since the advent of sonography, IHPS can be easily diagnosed and confirmed by abdominal sonography [2], [3]. The fluoroscopic upper gastrointestinal tract series also plays a role in IHPS diagnosis [4]. Ramstedt pyloromyotomy is currently a standard treatment of IHPS [5].

Studies have revealed that the incidence rate of IHPS varies among different ethnic groups around the world, as summarized in Table 1 [6], [7], [8], [9], [10], [11], [12], [13], [14], [15], [16], [17], [18], [19]. However, most of these studies were carried out in Western countries. To our limited knowledge, the epidemiology of IHPS has rarely been investigated in either ethnic Chinese populations or in Asian countries. The population of Taiwan, an island country located in Southeastern Asia, mainly consists of ethnic Chinese people. Taiwan has a National Health Insurance (NHI) system that covers more than 99% of the population. The NHI database provides an opportunity to conduct a population-based epidemiological study of IHPS.

**Table 1:** Comparison of the incidence and male-to-female rate of pyloric stenosis at different times and in different parts of the world.

Year studied	Authors	Area	No. of Cases	Incidence per 1000 live births	M/F rate	Trend
1968-1982	Lammer et al.	Atlanta, USA	518	1.332.90 (White, M)0.61 (Black, M)0.68 (White, F)0.17 (Black, F)	4.44∶1	NA
1971–1984	Hitchcock et al.	Western Australia	602	1.4–2.9	4.9∶1	Increasing
1974–1980	Knox et al.	Central Scotland, UK	1176	2.1–3.5	3.1–4.4∶1	Increasing
1976–19781986–1988	Tam et al.	Mersey, UK		1.542.22	3.55∶18.35∶1	Increasing
1981–2004	Sommerfield et al.	Scotland, UK	4950	4.4–1.4	NA	Declining
1983–1988	Schechter et al.	California, USA	1963	1.92.4 White1.8 Hispanic,0.7 Black0.6 Asian		
1983–1990	Applegate et al.	New York, USA	2304	2.4–1.7	4.31∶1	Declining
1987–1996	Hedback et al.	Sweden	2157	2.7–0.85	4.15∶1	Declining
1993–19961997–2000	To et al.	Ontario, North America	1918	1.81–1.481.55–1.98	4∶1	DecliningIncreasing
1999–2002	Wang et al.	Texas, USA	2747	2.14 White2.49 Hispanic-US1.58 Hispanic-F0.83 Blacks0.18 Chinese0.44 Vietnamese0.59 Asian Indians0.43Filipinos		
**1997–2007**	**Leong et al.(current study)**	**Taiwan**	**1077**	**0.39**	**4.3∶1**	**Declining**

F indicates female, M indicates male, NA indicates not available, UK indicates United Kingdom, USA indicates United States America, Hispanic-US indicates US-born Hispanic, Hispanic-F indicates foreign-born Hispanic.

Two environmental factors, including breastfeeding and maternal smoking, are thought to influence the incidence of IHPS [9], [13], [19], [20], [21], [22]. Babies born to smoking mothers have a twofold increased risk of being diagnosed with IHPS over babies born to nonsmoking mothers [21]. One study has shown that breastfeeding is a protective factor for IHPS [13], [22]. However, another study showed no link between breastfeeding and the incidence of IHPS [11].

The objectives of this study were (1) to describe the epidemiological characteristics of IHPS including the incidence, gender ratio, seasonal variation, age of diagnosis, and length and cost of hospitalization in Taiwanese children diagnosed with IHPS between 1997 and 2007 and (2) to study the possible relationship between the annual incidence of IHPS and two environmental factors, breastfeeding and maternal smoking.

## Materials and Methods

### Enrollment criteria of cases and data sources

The cases of IHPS for the present study were obtained from a National Health Insurance (NHI) claims database and selected based on the International Classification of Diseases, Ninth Revision, Clinical Modification (ICD-9-CM) code. Because Ramstedt pyloromyotomy is widely accepted as a standard treatment of IHPS, we selected all cases aged less than one year old, who were hospitalized at some point between January 1997 and December 2007 and were diagnosed with both ICD-9-CM 750.5 (for IHPS) and ICD-9-CM 43.3 (for positive pyloromyotomy). To meet the regulations of the Personal Electronic Data Protection Law of Taiwan, the initial identification numbers (ID) of all patients and hospitals in this database were converted to a new code to prevent recognition.

### Incidence by age and gender, hospitalization stay and cost

The annual incidence of IHPS was calculated using case numbers divided by total live births during the same year, and the value was expressed as number per 1,000 live births. The national life statistics on live births were obtained from the Department of Statistics, Ministry of the Interior, Taiwan [23]. The ratio of male-to-female live births was approximately 1.1: 1 from 1997 to 2007. This slight gender imbalance has been taken into consideration in all calculations of the male-to-female incidence ratio of IHPS. We also analyzed these hospitalizations by age, gender, month of hospitalization and year. The time from birth until treatment by pyloromyotomy, length of hospital stay and costs of IHPS were analyzed as well. Data on breastfeeding rates were obtained from the Bureau of Health Promotion, Department of Health, Taiwan [24]. The data were based on an annual sampling survey and contained two categories: exclusively breastfeeding rate and breastfeeding plus bottle feeding rate in the first month after delivery.

### Season definition

The four seasons were defined as winter (November, December and January), spring (February, March and April), summer (May, June and July) and autumn (August, September and October). The birth month was defined by the birth dates of infants, and the admission month was defined by the admission date of the hospitalization for IHPS management. The live birth numbers were almost equal among the four seasons from 2000 to 2007.

### Ethics approval

The Ethics Review Board of our institute has approved the study protocol. Because this is a secondary data analysis, no informed consent is needed.

### Statistical analysis

Statistical analysis was performed by using SPSS software (version 16.0 for Windows, Apache Software Foundation, U.S.A.). The difference in IHPS incidence between the two genders and among the four seasons was compared using a x^2^ test. A time trend analysis was performed to analyze the annual incidence of IHPS for two periods, 1997–2001 and 2002–2007. The correlation between IHPS incidence and breastfeeding rate, including exclusive breastfeeding and breastfeeding plus bottle feeding, was tested by linear regression. The analysis data are shown as mean ± standard deviation (SD) or median. A *P* < 0.05 was considered to be statistically significant.

## Results

### Case number and age at diagnosis

**Figure 1:**
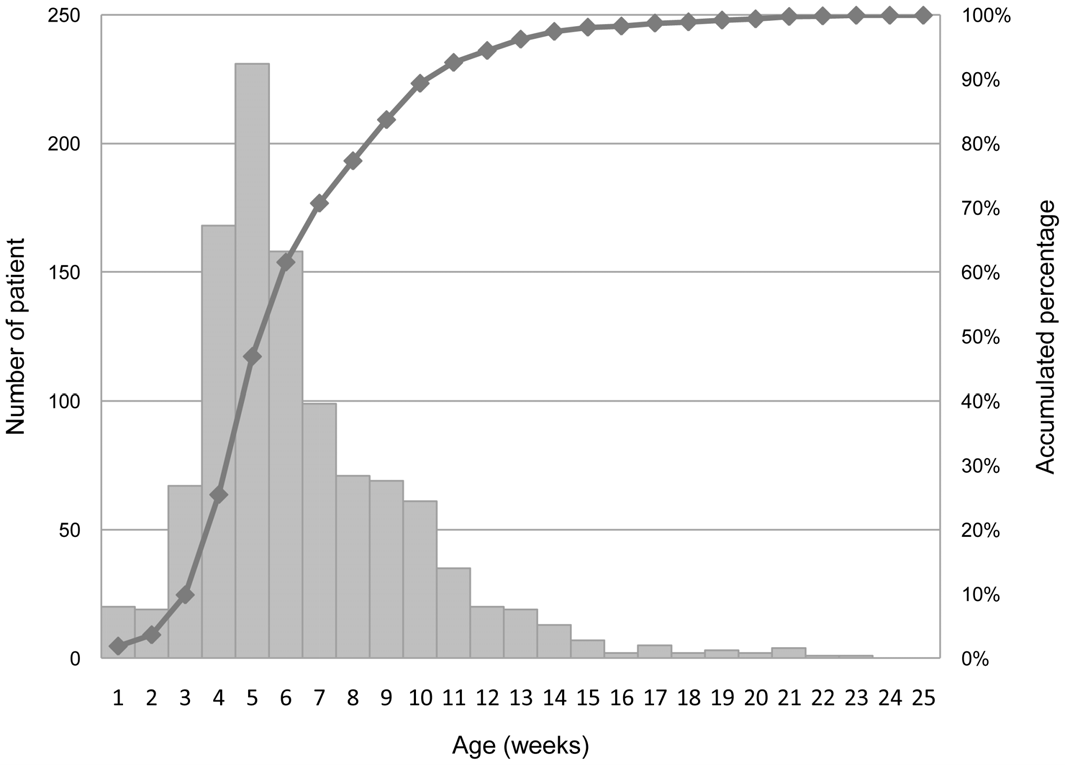
Case number and accumulated percent of cases by weeks of life at diagnosis between 1997 and 2007 in Taiwan.

A total of 1077 children under the age of one year old met the criteria for IHPS and pyloromyotomy and were hospitalized between 1997 and 2007. Among them, 889 (82.5%) cases were male. The mean ± standard deviation of diagnostic age was 43.1±2.4 days old (6.15±3.37 weeks old), with a median age of 37 days and interquartile age of 28 to 54 days old. The mean age of IHPS in males and females was 42.7±2.2 days and 44.9±2.9 days, respectively. The case numbers peaked at 5 weeks, and the accumulated percentage of cases reached 89.3% by the 10^th^ week of age as shown in Figure 1.

### Incidence and gender difference

Male children had a statistically significant higher incidence risk of IHPS diagnosis than female children (0.62 vs. 0.14 per 1,000 live births, respectively; *P* < 0.05). The male-to-female incidence rate ratio showed an increasing trend from 3.03 in the first two weeks of life to 8.94 in the 8^th^ through the 10^th^ weeks of life, and the overall male-to-female rate ratio was 4.30 as shown in Table 2.

**Table 2:** Case numbers of pyloromyotomy in the two genders and the male-to-female rate ratio in Taiwanese children between 1997 and 2007.

Age (week)	Male	Female	Male/Female ratio*
0∼2	30	9	3.03
2∼4	191	44	3.95
4∼6	323	65	4.52
6∼8	145	25	5.27
8∼10	118	12	8.94
10∼12	41	14	2.66
>12	41	19	1.96
**Total**	**889**	**188**	**4.30**

*The live birth numbers of male and female infants were approximate 1.1: 1 in each age group. So, the male/female ratio was the case number rates further divided by 1.1.

### Annual incidence and feeding practices

**Figure 2:**
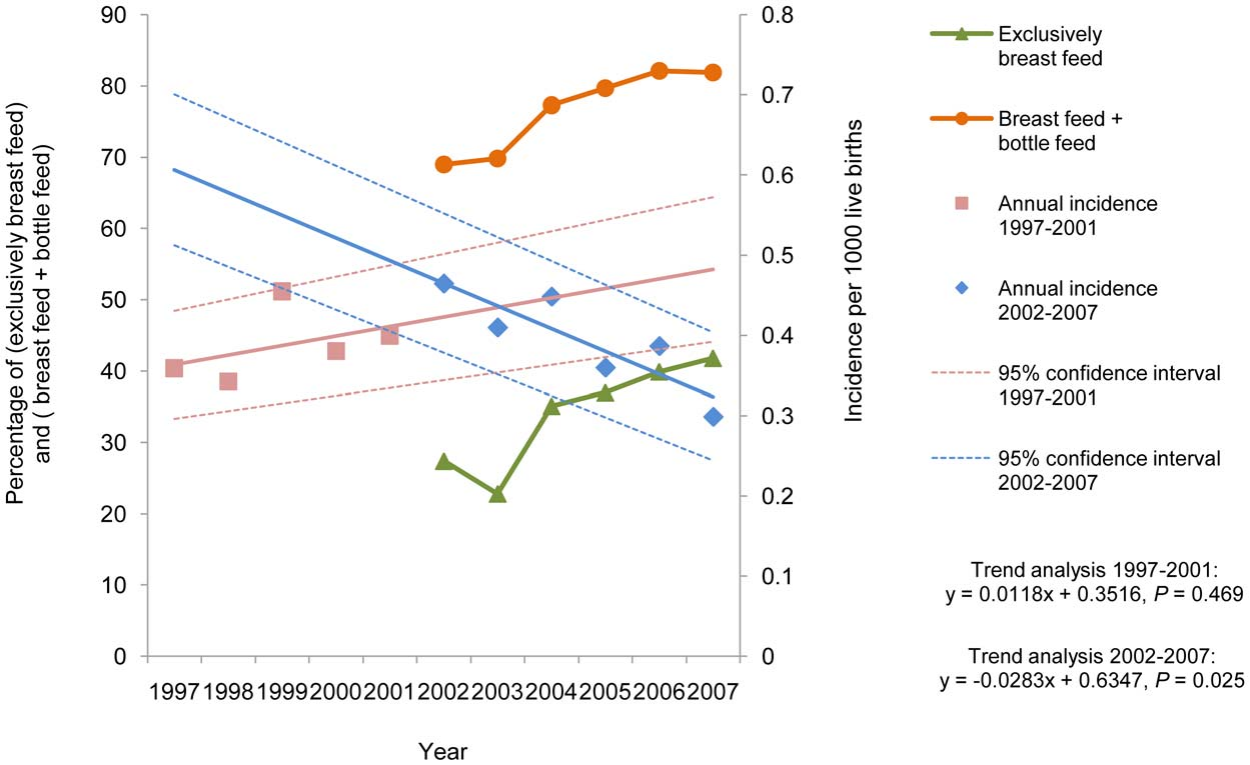
Annual incidence rate (per 1,000 live births) of pyloromyotomy among 1077 cases occurring between 1997 and 2007 in Taiwan.

The annual incidence of IHPS ranged from 0.30 to 0.47 per 1000 live births, with a mean incidence of 0.39 per 1000 live births. The time trend analysis showed a slowly increasing trend between 1997 and 2001 (*P* = 0.469), but a significant declining trend (*P* = 0.025) between 2002 and 2007 as shown in Figure 2. Concurrently, there were significant increasing trends for both exclusive breastfeeding (*P* = 0.014) and breastfeeding plus bottle feeding (*P* = 0.004) between 2002 and 2007. Moreover, the linear regression analysis showed an inverse correlation between the annual incidence of IHPS and breastfeeding rates between 2002 and 2007. The correlation coefficients for exclusive breastfeeding and breastfeeding plus bottle feeding were -0.653 (*P* = 0.116) and -0.705 (*P* = 0.118), respectively.

### Seasonal distribution

Between 2000 and 2007, the IHPS incidence of admission month by season was 0.433 per 1,000 live births in spring, 0.406 per 1,000 live births in summer, 0.347 per 1,000 live births in autumn and 0.394 per 1,000 live births in winter (*P* = 0.206). The IHPS incidence of birth month by season was 0.371 per 1,000 live births in spring, 0.388 per 1,000 live births in summer, 0.372 per 1,000 live births in autumn and 0.413 per 1,000 live births in winter (*P* = 0.708). Thus, the difference in incidence among the four seasons was not statistically significant either for admission month or birth month.

### Length of Hospitalization and Medical Expenses

The mean and median length of hospital stay was 8.28±7.10 and 7 days, respectively. The interquartile length of hospitalization stay was 5–9 days. The mean medical expense per person was $2000 USD (United States Dollar).

## Discussion

This present study was a population-based epidemiological analysis of IHPS in ethnic Chinese people. The studied population had an IHPS incidence rate of 0.39 per 1000 live births. These results are similar to those seen in previous studies (summarized in Table 1) that have shown that Asian populations have a lower incidence of IHPS than white populations [6], [7], [8], [10], [11], [12], [14], [15], [16], [17], [18], [19], [25]. The incidence rate of IHPS varies from 0.17 to 4.4 per 1000 live births among different populations around the world [6], [7], [8], [9], [10], [11], [12], [13], [14], [15], [16], [17], [18], [19]. One study in the United Sates found a generally lower incidence in Asian populations of differing backgrounds such as Chinese (0.18 per 1000 live births), Vietnamese (0.44 per 1000 live births), Asian Indians (0.59 per 1000 live births) and Filipinos (0.43 per 1000 live births) when compared to white populations [25]. Ethnic differences were also found in the Schechter study, with the highest incidence found in whites (2.4 per 1000 live births), in contrast to Hispanics (1.8 per 1000 live births) and blacks (0.7 per 1000 live births). The lowest incidence was found in Asians (0.6 per 1000 live births), as summarized in Table 1 [15]. Our results were consistent with these studies and confirmed that Chinese people in Asia have a much lower incidence of being diagnosed with IHPS than whites in Western countries [6], [7], [8], [10], [11], [12], [14], [15], [16], [17], [18], [19], [25].

The results of previous population-based cohort studies highly suggest that IHPS is a heritable disease with strong familial aggregation [26]. Therefore, the racial differences between IHPS incidence might be explained by genetic predisposition [26], [27]. In addition, other IHPS predisposing risk factors have also been proposed including birth rank [6], [9], [18], social class [6], [28], seasonal variation [6], [28], [29], [30], maternal smoking [21], feeding practices [9], [13], [20], [22], birth weight [31], blood group [32] and exposure to drugs such as erythromycin [33], [34]. In a study by Sorensen et al, 35 IHPS cases occurred among 16,725 births born to mothers who smoked (an incidence of 0.2%) compared to 43 cases among 41,271 births who born to mothers who did not smoke (an incidence of 0.1%) [21]. We compared the rates of maternal smoking in Taiwanese women with Western women to examine its possible association with the occurrence of IHPS. In Taiwan, the smoking prevalence rate for child-bearing women was only 4% [35], which is similar to other Asian countries but is much lower than the maternal smoking rates of Western developed countries, according to the statistics of the World Health Organization [36]. The correlation between the lower prevalence of maternal smoking and a lower incidence of IHPS in Taiwan may suggest that there is an association between maternal smoking and IHPS. However, this study was a secondary data analysis and lacked detailed information on rates of maternal smoking. The association should be cautiously interpreted.

Since 1998, to encourage breastfeeding, Taiwan has had a “Baby-Friendly Program Initiative” which asks hospitals to set up a friendly environment for mothers to breastfeed. Since 2001, the program has been a mandatory component of hospital accreditation, and the number of accredited hospitals increased from 58 in 2002 to 94 in 2007 [24]. Recent statistics also showed that the exclusive breastfeeding rate during the first month of the child's life dramatically increased from 27.4% in 2002 to 41.8% in 2007 [24]. For breastfeeding plus bottle feeding, the rates increased as well, as shown in Figure 2. Concurrent with the trend of increasing breastfeeding rates, this study found a decreasing trend of IHPS beginning in 2002. The annual incidences of IHPS and breastfeeding rates showed an inverse correlation between 2002 and 2007; however, the difference was not statistically significant, most likely due to the short observational period. The association between the initiation of a breastfeeding campaign in Taiwan and the decline in the incidence of IHPS might suggest that breastfeeding protects against IHPS [9], [13], [22], though the decline of IHPS incidences may be also explained by other factors. Two case-control studies have found that bottle feeding groups have a higher risk of developing IHPS than breastfeeding groups [13], [20].

Several mechanisms have been suggested to explain the possible protective effect of breastfeeding in IHPS. For example, breast milk promotes better gastric emptying due to its lower osmolarity [37]. It also has higher levels of pylorus-relaxing hormones such as vasoactive intestinal peptide [20]. In contrast, bottle feeding may delay gastric emptying [37], [38], and formula milk may increase the plasma gastrin concentration. Increases in plasma gastrin may be associated with pylorospasm and pyloric hypertrophy [20], [39]. Consistent with the results of previous studies, the present study showed a predominance of IHPS among male children [6], [7], [8], [10], [11], [12], [14], [15], [16], [17], [18], [19]. Moreover, this study also showed that the male-to-female ratio was a dynamic ratio instead of a fixed value among different age groups. The ratio increased gradually from the first two weeks to the 8^th^ through the 10^th^ weeks and then decreased rapidly as shown in Table 2. The reason for the gradual increase of the male-to-female ratio may be due to the differential onset time of IHPS between the two sexes, as the mean age of IHPS onset was older in females than in males (6.42 weeks *vs*. 6.10 weeks, respectively). This dynamic ratio change was also observed for childhood intussusception rates [40]. The male-to-female ratio decreased after 12 weeks of age. The decrease was probably due to the fewer number of overall cases, which inhibited us from making any strong inferences with regard to older children. The mean age of diagnosis of IHPS was 43 days, similar to previous studies that found the mean to be approximately 40 days [25], [41]. The accumulated percentage of IHPS reached almost 90% by the 10^th^ week of age, which was also consistent with previous studies [15], [18].

The seasonal variation of the occurrence of IHPS has varied in different studies in the past [6], [28], [29], [30]. One previous study suggested that a higher incidence of IHPS was found in infants born in the third quarter (July–September) [28]. Dodge et al. reported that more IHPS infants were born in winter months, and the peak admission season was during the winter. However, seasonal variation was not found by Rasmussen et al. and Lemessa et al. [29], [30]. Our study did not show significant differences among the four seasons, either for the admission month or the birth month. The mean length of hospital stay was 8.28 days, which was a bit longer than the 6.14 days found in a study by Gibb et al. [1].

### Conclusions

The incidence of IHPS was much lower in Taiwan, a country whose majority population is ethnic Chinese, compared to the rates observed in Caucasian populations in Western countries. Environmental factors such as breastfeeding and lower maternal smoking rates may contribute to the lower incidence of IHPS in Taiwan. Moreover, the male-to-female rate ratio of IHPS was found to be a dynamic ratio instead of a fixed value. No seasonal variation of IHPS was found in Taiwan.
